# L-Dopa Pharmacokinetic Profile with Effervescent Melevodopa/Carbidopa versus Standard-Release Levodopa/Carbidopa Tablets in Parkinson's Disease: A Randomised Study

**DOI:** 10.1155/2015/369465

**Published:** 2015-06-10

**Authors:** Fabrizio Stocchi, Laura Vacca, Paola Grassini, Stephen Pawsey, Holly Whale, Stefano Marconi, Margherita Torti

**Affiliations:** ^1^Institute of Neurology, IRCCS San Raffaele, Via della Pisana 235, 00163 Rome, Italy; ^2^Vernalis (R&D) Ltd., Reading Road, Winnersh, Berkshire RG41 5UA, UK; ^3^Chiesi Farmaceutici SpA, 43100 Parma, Italy

## Abstract

*Objectives.* To characterize the pharmacokinetic profile of levodopa (L-dopa) and carbidopa after repeated doses of the effervescent tablet of melevodopa/carbidopa (V1512; Sirio) compared with standard-release L-dopa/carbidopa in patients with fluctuating Parkinson's disease. Few studies assessed the pharmacokinetics of carbidopa to date. *Methods.* This was a single-centre, randomized, double-blind, double-dummy, two-period crossover study. Patients received V1512 (melevodopa 100 mg/carbidopa 25 mg) or L-dopa 100 mg/carbidopa 25 mg, 7 doses over 24 hours (Cohort 1), 4 doses over 12 hours (Cohort 2), or 2 doses over 12 hours in combination with entacapone 200 mg (Cohort 3). Pharmacokinetic parameters included area under the plasma-concentration time curve (AUC), maximum plasma concentration (*C*_max_), and time to *C*_max_ (*t*_max_). *Results.* Twenty-five patients received at least one dose of study medication. L-dopa absorption tended to be quicker and pharmacokinetic parameters less variable after V1512 versus L-dopa/carbidopa, both over time and between patients. Accumulation of L-dopa in plasma was less noticeable with V1512. Carbidopa exposure and interpatient variability was lower when V1512 or L-dopa/carbidopa was given in combination with entacapone. Both treatments were well tolerated. *Conclusions.* V1512 provides a more reliable L-dopa pharmacokinetic profile versus standard-release L-dopa/carbidopa, with less drug accumulation and less variability. This trial is registered with ClinicalTrials.gov NCT00491998.

## 1. Introduction

Levodopa (L-dopa) plus peripheral dopa-decarboxylase inhibitor (carbidopa or benserazide) is the mainstay of therapy in Parkinson's disease [1]. However, motor and nonmotor complications emerging during chronic treatment become increasingly more common with advancing disease [2, 3]. Complications, dyskinesias and response fluctuations, include end-of-dose wearing “off,” sudden “on”/“offs,” delayed time to “on,” and response failures [4]. Factors interfering with L-dopa delivery and absorption may induce motor fluctuations [5–8]. Thus, limitations in the physical properties and pharmacokinetics of L-dopa, poor solubility, low bioavailability, and short half-life, play an important role in the occurrence of fluctuations [9, 10].

In order to reach its site of absorption in the small intestine, orally administered L-dopa has to pass through the stomach and thus is reliant on gastric emptying, which is often delayed in Parkinson's disease patients due to the effects of the disease, its treatments, and dietary intake [11–14]. Gastric akinesia is common in advanced Parkinson's disease and may lead to rapid rises in plasma L-dopa concentrations when delayed gastric emptying results in an oral dose of L-dopa sitting in the stomach for some time before it passes into the duodenum where it is immediately absorbed [15]. This “dose-dumping” effect is hereafter referred to as “apparent accumulation.” Another problem that hinders optimal absorption of oral L-dopa is poor solubility when formulated as a tablet [15]. Compared with conventional tablets, L-dopa solutions pass more easily through the stomach thereby ensuring better absorption, but their use is limited by poor aqueous solubility and degradability of L-dopa in solution [16].

V1512 is an effervescent tablet formulation of L-dopa methyl ester (also known as melevodopa) and carbidopa. Melevodopa is approximately 250 times more soluble than L-dopa, allowing rapid and complete dissolution, and the resulting solution rapidly reaches the absorption site [17]. Furthermore, at physiological pH, melevodopa exists in the nonionized form whereas L-dopa exists in the ionized from, and the resulting greater lipophilicity of melevodopa enhances its absorption across the intestinal wall [17]. Studies performed in animals and humans have shown complete prehepatic hydrolysis to L-dopa and rapid intestinal absorption that is not influenced by gastric pH [16, 18–20]. These factors suggest that the onset of action of melevodopa should occur sooner compared with conventional oral L-dopa formulations, and this has been confirmed in clinical studies [21, 22].

The primary objective of this study was to characterize the pharmacokinetic profile of L-dopa after repeated doses of V1512 compared with standard-release L-dopa/carbidopa tablets in patients with fluctuating Parkinson's disease.

## 2. Methods

The study was conducted at the Institute for Research and Medical Care in San Raffaele, Rome, Italy, after approval by the Ethics Committee and was performed in accordance with the Declaration of Helsinki and Good Clinical Practice.

### 2.1. Patients

Eligible patients were aged >30 years with a body mass index (BMI) of 18.5–29.9 kg/m^2^ inclusive and a clinical diagnosis of Parkinson's disease according to the Brain Bank criteria [23]. Patients had motor performance fluctuations with >2 hours total daily “off” and ≥1 hour delay to “on” time with afternoon doses and were permitted to take other Parkinson's therapies (dopamine agonists, monoamine oxidase-B inhibitors) provided the doses had been stable for at least 2 weeks before study entry. Other comorbidities had been stable for at least 4 weeks and females were to be of nonchildbearing potential. Patients were informed of the objectives, procedures, and risks of study participation and gave written informed consent.

### 2.2. Study Design

In this single-centre, randomized, double-blind, double dummy, two-period, crossover study, subjects were randomized to treatment with molar equivalent doses of V1512 (Sirio, Chiesi Farmaceutici SpA, Parma, Italy) and then L-dopa/carbidopa (Sinemet 100/25; Merck Sharp & Dohme, Whitehouse Station, NJ, USA), or L-dopa/carbidopa and then V1512, during two 1-day treatment periods.

Following screening, eligible patients underwent a 2-week run-in period, in which patients received L-dopa/carbidopa 100/25 mg standard-release tablets at a frequency determined by the investigator and no other L-dopa therapies were permitted. In practice, most patients were already established on a suitable stable dose of L-dopa/carbidopa at recruitment and thus did not undergo a formal run-in period. In the double-blind phase, patients were assigned to one of three treatment cohorts based on the patient's usual dosing regimen: the first dose of study medication was administered to patients at 08:00 hours, and subsequent doses were administered every 2 hours for patients in Cohort 1 (total of 6 doses over 12 hours) and every 3 hours for patients in Cohorts 2 and 3 (4 doses over 12 hours (see Supplementary Table 1 available online at http://dx.doi.org/10.1155/2015/369465)). Levodopa was administered every 2 hours with the goal of obtaining a constant levodopa level and every 3 hours to mimic the most common clinical practice in patients with motor fluctuations. Entacapone was given to Cohort 3 every 4 hours for the same reason. Within each cohort, patients were randomized (computer-generated randomization with a block size of 4) to receive V1512 solution plus placebo tablets in the first treatment period followed by L-dopa/carbidopa tablets plus placebo solution in the second treatment period, or vice versa. Patients in Cohort 3 also received 200 mg entacapone (Comtan; Novartis, Orion Pharma, Basel, Switzerland) with each dose of active treatment. The treatment sequence allocation was concealed from the investigator. The two double-blind treatment periods were separated by a 2-day washout, during which patients received the same standardized regimen they had received during the run-in phase (or their usual Parkinson's treatment if they did not undergo a run-in period). Investigators at site were responsible for enrolling patients into the trial; a pharmacist was responsible for assignment of patients to treatment interventions and the study drug was administered by a study nurse. The active V1512 and placebo V1512 effervescent tablets were identical in appearance and produced solutions with the same appearance and taste. However, the placebo L-dopa/carbidopa tablet was slightly different in appearance to the active L-dopa/carbidopa tablet (in terms of markings) and thus, each tablet was placed in a narrow opaque tube for administration to the patient. The patient, investigator, Medical Monitor, and Clinical Monitor remained blinded, whereas the site pharmacist and the nurse who administered study medication had access to the randomization list.

Patients remained in the study unit during treatment days, treatment administration was supervised, and all were given standardized low-protein meals. Each dose of V1512 was a single effervescent tablet (125.6 mg melevodopa (equivalent to 100 mg L-dopa) and 25 mg carbidopa) dissolved in 150 mL water, which was ingested at the same time as a placebo tablet. During the other treatment period, an L-dopa/carbidopa standard-release tablet (containing 100 mg L-dopa and 25 mg carbidopa) was ingested at the same time as placebo solution (an effervescent placebo tablet dissolved in 150 mL water).

The occasional use of paracetamol (acetaminophen) was permitted during the two treatment days, but no other medications were allowed. All Parkinson's disease medications were discontinued from 20:00 hours on the day before dosing until 20:00 hours on the day of dosing. During the run-in period, patients were instructed to discontinue catechol-O-methyl transferase inhibitors (except those in Cohort 3).

### 2.3. Pharmacokinetic Analysis

Pharmacokinetic parameters for L-dopa and carbidopa were calculated from blood samples taken immediately before and at regular intervals (20 or 30 minutes) after dose administration during both study periods (Supplementary Table 1).

Blood samples were collected in sodium heparin Vacutainers, and plasma was obtained by cooled centrifugation at 3,000 rpm for 10 minutes. Plasma samples were stored at −60°C or −80°C until analysis. All samples were analysed using a validated analytical method (liquid chromatography tandem mass spectrometry). Validation studies demonstrated that the analytical method shows good linearity over the calibration range (regression correlation coefficient 0.9993) and has good accuracy (no more than two quality control (QC) samples at different levels fell outside the acceptance limits of ±15% of target, and in the case of dilutions; no more than one QC sample at the corresponding dilution level fell outside the acceptance limits of ±15% of target) and precision (percentage coefficient of variation (CV%) 2.6–6.6%). Back-calculated concentrations of the calibration lines showed excellent mean relative error, with deviations from the target of ≤1.0% for all L-dopa plasma calibrants, indicating good specificity. The limit of quantification for L-dopa and carbidopa was 10 and 25 ng/mL, respectively.

L-dopa and carbidopa pharmacokinetic parameters calculated included area under the plasma concentration time curve (AUC), maximum observed plasma concentration (*C*_max_), and time to *C*_max_ (*t*_max_). AUC was calculated using the linear or logarithmic trapezoidal methods when concentrations were increasing or decreasing, respectively. Pharmacokinetic analyses were performed on all patients with data available.

### 2.4. Safety

Safety and tolerability were assessed using adverse event (AE) monitoring, standard clinical laboratory tests (biochemistry, haematology and urinalysis), 12-lead electrocardiogram (ECG), and vital signs measurements (blood pressure and pulse).

### 2.5. Statistical Analysis

A proposed sample size of 27 patients was considered sufficient to evaluate possible differences between the L-dopa pharmacokinetic profiles of V1512 and L-dopa/carbidopa and is typical of exploratory pharmacokinetic studies. Pharmacokinetic parameters are summarized using descriptive statistics. Plots of individual L-dopa plasma concentrations are presented by treatment group.

## 3. Results

### 3.1. Patients

Subjects were recruited from November 2006 to November 2007. The last follow-up was performed on December 2007. Twenty-five patients were randomized and received ≥1 dose of study medication, with 21 patients completing the study; 23 patients were evaluated for pharmacokinetics (7, 8, and 8 patients in Cohorts 1, 2, and 3, resp.); 2 patients were excluded from these analyses because no relevant postdose pharmacokinetic data were collected (they withdrew prematurely due to withdrawal of consent and difficulty obtaining venous access). The other two patients who did not complete the study (both in Cohort 1) were lost to follow-up and withdrew consent. Protocol violations were recorded for 10 patients but were minor and did not affect the integrity or interpretation of the data. Although the target of 27 patients completing both treatment periods was not met, the number of patients analysed was considered sufficient to assess differences between L-dopa pharmacokinetic profiles of V1512 and L-dopa/carbidopa.

Demographic data were generally representative of patients with moderate-to-severe Parkinson's disease. Cohorts were well matched for daily “off” time and disease stage (Supplementary Table 1). However, the baseline clinical characteristics were less severe than would generally be expected for patients with moderate-to-severe Parkinson's disease (mean total daily “off” time was approximately 4 hours and only one patient (4%) had disease severity of Stage 4 according to Hoehn and Yahr staging [24]).

### 3.2. Pharmacokinetics of L-Dopa

**Figure 1:**
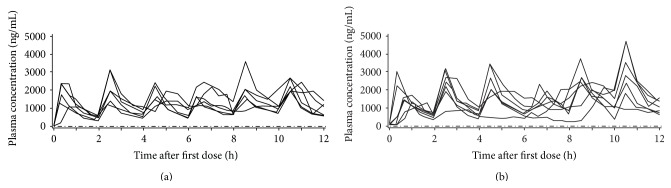
L-dopa plasma concentrations for patients in Cohort 1 (dose administration every 2 hours) receiving V1512 solution (a) or L-dopa/carbidopa 100/25 mg standard-release tablets (b). All patients with data available for the pharmacokinetic analyses were included; patients with significant violations that may have invalidated or biased the results of the pharmacokinetic evaluations were excluded. –·–·–· = lower limit of quantification (10 ng/mL).

**Figure 2:**
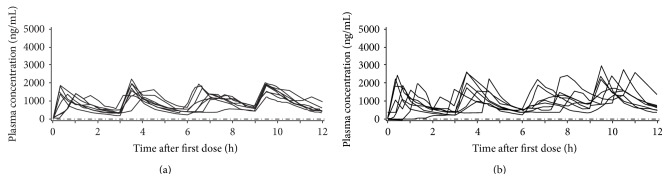
L-dopa plasma concentrations for patients in Cohort 2 (dose administration every 3 hours) receiving V1512 solution (a) or L-dopa/carbidopa 100/25 mg standard-release tablets (b). All patients with data available for the pharmacokinetic analyses were included; patients with significant violations that may have invalidated or biased the results of the pharmacokinetic evaluations were excluded. –·–·–· = lower limit of quantification (10 ng/mL).

**Figure 3:**
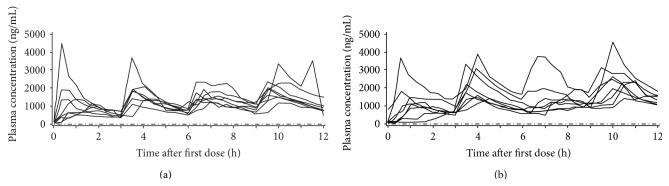
L-dopa plasma concentrations for patients in Cohort 3 (dose administration every 3 hours) receiving V1512 solution plus entacapone 200** **mg (a) or L-dopa/carbidopa 100/25** **mg standard-release tablets plus entacapone 200** **mg (b). All patients with data available for the pharmacokinetic analyses were included; patients with significant violations that may have invalidated or biased the results of the pharmacokinetic evaluations were excluded. –·–·–· = lower limit of quantification (10 ng/mL).

L-dopa pharmacokinetic parameters for Cohorts 1, 2, and 3 are summarized in Figures 1, 2, and 3. (Supplementary Tables 2, 3, and 4).

Following V1512 and L-dopa/carbidopa administration, L-dopa was rapidly absorbed, with median *t*_max_ values in Cohorts 1 and 2 ranging from 0.5 to 1.0 hour after dose. However, the variation in values between patients was narrower with V1512 than with L-dopa. Furthermore, *t*_max_ was shorter with V1512 for the first morning dose and the first afternoon dose by approximately 0.3 hours. Maximum plasma concentrations of L-dopa appeared to occur slightly later when V1512 and L-dopa/carbidopa were coadministered with entacapone compared with each treatment administered alone. Again, the median *t*_max_ was shorter for all four doses of V1512 versus L-dopa, with the difference for the first afternoon dose (dose 3) being nearly 1 hour. The range of median *t*_max_ values also tended to be narrower with V1512, particularly for dose 3 where the difference in the range of values was 1.3 hours.

In all three cohorts, there was apparent accumulation of L-dopa in the plasma (as judged by higher concentrations in the evening compared with the morning) following dosing with V1512, but the effect was more noticeable with L-dopa/carbidopa.

From dose 1 to the final dose in Cohorts 1, 2, and 3, respectively, mean AUC_0–*t*_ values increased by 51%, 50%, and 85% with V1512 and by 92%, 93%, and 150% with L-dopa/carbidopa. From dose 1 to the final dose in Cohorts 1, 2, and 3, respectively, mean *C*_max_ values increased by 30%, 14%, and 52% with V1512 and by 64%, 23%, and 99% with L-dopa/carbidopa. Furthermore, all three cohorts showed a higher exposure after the L-dopa/carbidopa final dose; this was less apparent after the V1512 final dose.

Following doses 1 and 3 in Cohort 3 and doses 1 and 4 in Cohort 1, mean AUC_0–*t*_ values were higher following V1512 administration than after L-dopa/carbidopa. These higher values indicate that V1512 was more effective at delivering L-dopa at these times (early morning and early afternoon). Over the entire dosing period, L-dopa exposure was slightly lower after V1512 compared to L-dopa/carbidopa administration in all three cohorts (AUC was approximately 8% lower and *C*_max_ was up to 26% lower). Coadministration of V1512 or L-dopa/carbidopa with entacapone in Cohort 3 resulted in higher exposure (AUC_0–*t*_) to L-dopa after each dose compared to each treatment administered alone (Cohort 2).

Across the six dosing intervals in Cohort 1, L-dopa maximal levels were less variable following V1512 administration, with mean *C*_max_ values differing by 1.4-fold compared with 1.7-fold following L-dopa/carbidopa administration. Across the four dosing intervals in Cohorts 2 and 3, maximal levels of L-dopa were also less variable following V1512 administration, with mean *C*_max_ values differing by 1.3-fold and 1.5-fold, respectively, compared with 1.5-fold and 2.0-fold, respectively, following L-dopa/carbidopa administration. Similar reduced variability in AUC was also observed for V1512 compared with L-dopa/carbidopa across the dosing intervals in all three cohorts.

The interpatient variability for L-dopa following dosing with V1512 alone in Cohorts 1 and 2 was moderate, with geometric CV% values ranging from 17 to 33% for AUC and 13 to 41% for *C*_max_ across all doses. Variability was higher after dosing with L-dopa/carbidopa alone, with CV% values ranging from 20 to 53% for AUC and 21–72% for *C*_max_ across all doses. When V1512 and L-dopa/carbidopa were coadministered with entacapone, variability was higher than for each administered alone in Cohort 2, with AUC and *C*_max_ CV% values, respectively, ranging from 23 to 45% and 20 to 71% for V1512 and 29 to 74% and 35 to 60% for L-dopa/carbidopa. The corresponding ranges of AUC and *C*_max_ CV% values in Cohort 2 were 17–30% and 13–41% for V1512 and 20–53% and 25–34% for L-dopa/carbidopa. The reduced variability in L-dopa plasma concentrations during V1512 compared to L-dopa/carbidopa treatment can be seen for Cohorts 1, 2, and 3 in Figures 1, 2, and 3, respectively.

### 3.3. Pharmacokinetics of Carbidopa

Accumulation of carbidopa was evident in all 3 cohorts, but in contrast to that observed with L-dopa, exposure to carbidopa was lower following combination treatment of V1512 or L-dopa/carbidopa with entacapone (Cohort 3) compared with each treatment administered alone (Cohort 2). However, this difference was not clinically relevant.

Interpatient variability in carbidopa plasma concentrations was similarly high in Cohorts 1 and 2 but was slightly lower in Cohort 3 (i.e., when given with entacapone). *t*_max_ values also varied quite substantially and there were no meaningful differences between V1512 and L-dopa/carbidopa in any cohort.

### 3.4. Safety

V1512 was well tolerated during the study, and only a single treatment-emergent AE (contusion) was recorded. No deaths, serious AEs, discontinuations due to AEs, or other significant AEs were reported during the study. No safety concerns were raised from clinical laboratory evaluations, vital signs measurements, or ECG data.

## 4. Discussion

The results show that L-dopa was rapidly absorbed following both V1512 and L-dopa/carbidopa dosing when treatments were administered alone. Although the between-group difference did not reach statistical significance, absorption of V1512 absorption tended to be more rapid than that of L-dopa/carbidopa. This is not surprising given that V1512 is administered as a solution and so there is no delay associated with the need for dissolution prior to absorption. The pharmacodynamic advantage of earlier absorption has been demonstrated in a number of studies in which L-dopa methyl ester preparations were associated with approximately 12-minute improvement in onset of action compared with other L-dopa/dopa decarboxylase inhibitors [17, 21]. In general, *C*_max_ and AUC values tended to be higher for the later doses, particularly the final dose, because of delayed absorption of the previous dose (apparent accumulation). However, there was less apparent accumulation of L-dopa in plasma following administration of V1512 compared to L-dopa/carbidopa, reflecting more reliable absorption of the previous V1512 dose. In all three cohorts, measured V1512 pharmacokinetic parameters were generally less variable than L-dopa/carbidopa, both throughout the 12-hour dosing period and between patients within a cohort. In particular, L-dopa plasma concentrations during the early afternoon and evening were more evenly maintained with both 2- and 3-hourly V1512 dosing regimens compared with L-dopa/carbidopa.

Although overall exposure to L-dopa was lower over the entire dosing period with V1512 compared to L-dopa/carbidopa treatment, this is unlikely to reduce efficacy and is probably due to a reduction in “rebound” absorption resulting from failure to absorb previous doses fully. In fact, the greater reproducibility of plasma L-dopa concentrations seen after V1512 administration may limit side effects such as dyskinesia without having to reduce the dose and/or increase dose frequency. As expected, coadministering either V1512 or L-dopa/carbidopa with entacapone resulted in higher L-dopa exposure than when either formulation was administered alone. This finding is consistent with the known ability of entacapone to increase the AUC of L-dopa by inhibiting its metabolism [25].

The observation that V1512 appeared to be more effective than L-dopa/carbidopa at delivering L-dopa in the early morning and early afternoon is promising, because L-dopa delivery at these time points is known to be problematic in patients with advanced Parkinson's disease [10, 22, 24, 26]. This finding, along with the reduced interpatient variability seen after administration of V1512, indicate that V1512 administration is associated with reduced fluctuation in L-dopa exposure in patients with Parkinson's disease. We have previously demonstrated a clear trend towards improved overall control of the motor complications of Parkinson's disease with V1512 compared to L-dopa/carbidopa [27], and it is probable that these clinical advantages result from the more reliable and reproducible absorption of L-dopa from V1512.

In this study there were no obvious differences in safety profile between the two treatments, both of which were well tolerated by this group of L-dopa-experienced patients.

One of the strengths of this study is that it is one of the few to examine the pharmacokinetics of carbidopa. The main limitation of this study is the small number of subjects. Replication of the results in a larger group of patients would be beneficial, as would assessment of differences between the treatments in clinical effects.

In summary, the results of this study indicate that V1512 has a more reliable L-dopa pharmacokinetic profile versus standard-release L-dopa/carbidopa, with less apparent drug accumulation, less variability throughout the dosing period and between patients, and more effective L-dopa delivery after the early morning (08:00) and early afternoon (14:00) doses. Although delayed gastric emptying can impair levodopa absorption in PD patients, drug delivery via an effervescent formulation of levodopa appeared to be slightly more reliable and faster acting than standard preparation. Moreover, effervescent V1512 formulation has the advantage that it can be easily administered through a nasogastric tube in postsurgical patients or those with swallowing problems.

## Supplementary Material

Supplementary Table 1: Dosing schedule and post-dose blood draw times to obtain samples for pharmacokinetic analysis.
Supplementary Table 2: Demographic and baseline characteristics for all randomized patients.
Supplementary Table 3: Pharmacokinetic parameters for L-dopa: Cohort 1
Supplementary Table 4: Pharmacokinetic parameters for L-dopa: Cohort 2

## Key Points

V1512 has a more reliable L-dopa pharmacokinetic profile versus standard-release L-dopa/carbidopa, with more effective L-dopa delivery in the early morning and afternoon.
V1512 pharmacokinetic parameters were generally less variable than L-dopa/carbidopa, both over time and between patients.
These results indicate that V1512 administration results in reduced fluctuation in L-dopa exposure in patients with Parkinson's disease.
