# Supplementary material for: L-Dopa Pharmacokinetic Profile with Effervescent Melevodopa/Carbidopa versus Standard-Release Levodopa/Carbidopa Tablets in Parkinson's Disease: A Randomised Study
Source: Parkinsons Dis. 2015 Jun 10;2015:369465. doi: 10.1155/2015/369465 (PMC4478358; doi:10.1155/2015/369465)
Supplement: Supplementary file 1 — Supplementary Table 1: Dosing schedule and post-dose blood draw times to obtain samples for pharmacokinetic analysis. Supplementary Table 2: Demographic and baseline characteristics for all randomized patients. Supplementary Table 3: Pharmacokinetic parameters for L-dopa: Cohort 1 Supplementary Table 4: Pharmacokinetic parameters for L-dopa: Cohort 2 [file 369465.f1.pdf]

Appendix A – Supplementary Tables

SUPPLEMENTARY TABLE 1: Dosing schedule and post-dose blood draw times to obtain samples for pharmacokinetic analysis.

	Time of dosing – Cohort 1 (hours)					
	08:00	10:00	12:00	14:00	16:00	18:00
Post-dose sampling times (minutes)	0, 20, 40, 60, 80, 100	0, 30, 60, 90	0, 30, 60, 90	0, 20, 40, 60, 80, 100	0, 30, 60, 90	0, 30, 60, 90, 120

	Time of dosing – Cohorts 2 and 3 (hours)			
	08:00	11:00	14:00	17:00
Post-dose sampling times (minutes)	0, 20, 40, 60, 80, 100, 120, 140, 160	0, 30, 60, 90, 120, 150	0, 20, 40, 60, 80, 100, 120, 140, 160	0, 30, 60, 90, 120, 150, 180

SUPPLEMENTARY TABLE 2: Demographic and baseline characteristics for all randomized patients.

	**Cohort 1 (n=7)**	**Cohort 2 (n=10)**	**Cohort 3 (n=8)**	**Total (n=25)**
Age, years	67.7 ± 6.78	69.8 ± 7.91	56.8 ± 14.58	65.0 ± 11.46
	(57-79)	(57-81)	(40-77)	(40-81)
Male, n (%)	5 (71.4)	5 (50.0)	4 (50.0)	14 (56.0)
Female, n (%)	2 (28.6)	5 (50.0)	4 (50.0)	11 (44.0)
Caucasian, n (%)	7 (100.0)	10 (100.0)	8 (100.0)	25 (100.0)
Height, cm	166.0 ± 4.04	167.1 ± 6.45	169.3 ± 12.43	167.5 ± 8.16
	(160-172)	(160-181)	(150-192)	(150-192)
Weight, kg	67.9 ± 6.84	68.0 ± 9.71	72.8 ± 16.60	69.5 ± 11.52
	(60-80)	(55-87)	(47-105)	(47-105)
BMI, kg/m^2	24.64 ± 2.020	24.41 ± 2.868)	25.11 ± 3.204	24.70 ± 2.682
	(21.4-27.1)	(19.6-27.6)	(20.8-28.5)	(19.6-28.5)
Daily 'off' time, hours	3.8 ± 1.68	4.1 ± 1.01	4.4 ± 1.05	4.1 ± 1.22
	(2-6)	(3-6)	(3-6)	(2-6)
Time since diagnosis of PD,	11.57 ± 3.867	10.83 ± 6.663	4.08 ± 2.408	8.88 ± 5.784
years*	(4.0-15.0)	(3.7-24.0)	(0.7-8.0)	(0.7-24.0)
Hoehn & Yahr staging, n (%)				
1	0	0	0	0
2	4 (57.1)	5 (50.0)	4 (50.0)	13 (52.0)
3	3 (42.9)	4 (40.0)	4 (50.0)	11 (44.0)
4	0	1 (10.0)	0	1 (4.0)
5	0	0	0	0

Values are mean ± standard deviation (range), or number of patients (%).
BMI = body mass index; PD = Parkinson's disease. *Calculated as the number of years from the date of diagnosis to the date of Visit 1.

SUPPLEMENTARY TABLE 3: Pharmacokinetic parameters for L-dopa: Cohort 1 (including all patients with data available for the pharmacokinetic analyses and excluding patients with significant violations that may have invalidated or biased the results of the pharmacokinetic evaluations).

	**Cohort 1: 2-hourly dosing regimen**	
	**V1512 (n=6)***	**L-dopa/carbidopa (n=7)**
$AUC_{0\text{-}t}$, ng • h • mL^{-1}		
Dose 1	1871 (32.7)	1706 (28.7)
Dose 2	2306 (32.8)	2505 (32.9)
Dose 3	2313 (19.2)	2480 (53.1)
Dose 4	2426 (29.9)	1946 (46.3)
Dose 5	2499 (27.4)	3151 (23.0)
Dose 6	2818 (21.8)	3275 (45.5)
$AUC_{0\text{-}12h}$, ng • h • mL^{-1}	14791 (24.0)	15887 (24.2)
$AUC_{0\text{-}6h}$, ng • h • mL^{-1}	6653 (25.9)	6880 (35.2)
$AUC_{6\text{-}12h}$, ng • h • mL^{-1}	8095 (25.2)	8918 (19.6)
C_{max}, ng/mL		
Dose 1	1792 (35.1)	1624 (36.6)
Dose 2	2029 (39.3)	2190 (45.2)
Dose 3	1835 (22.6)	1990 (72.3)
Dose 4	1700 (27.7)	1524 (51.1)
Dose 5	1898 (39.9)	2531 (20.6)
Dose 6	2329 (13.4)	2662 (58.3)
$C_{max0\text{-}12h}$, ng/mL	2674 (20.0)	3350 (26.6)
t_{max}, h		
Dose 1	0.333 (0.233-0.667)	0.667 (0.317-1.33)
Dose 2	0.500 (0.417-0.500)	0.500 (0.467-1.50)

Dose 3	0.550 (0.500-1.00)	0.500 (0-1.00)
Dose 4	0.625 (0.333-1.00)	1.00 (0-1.98)
Dose 5	0.500 (0.467-1.00)	0.500 (0.467-1.50)
Dose 6	0.500 (0.500-1.00)	0.500 (0-0.500)

Geometric mean (percentage coefficient of variation [CV%]) values shown for AUC and C_{max}; median (range) values shown for t_{max}.
AUC = area under the curve; C_{max} = maximum observed plasma concentration; t_{max} = time to C_{max}.
*One patient in this cohort prematurely withdrew from the study before receiving V1512.

SUPPLEMENTARY TABLE 4: Pharmacokinetic parameters for L-dopa: Cohort 2 (including all patients with data available for the pharmacokinetic analyses and excluding patients with significant violations that may have invalidated or biased the results of the pharmacokinetic evaluations).

	**Cohort 2: 3-hourly dosing regimen**	
	**V1512 (n=8)**	**L-dopa/carbidopa (n=8)**
AUC_{0-t}, ng • h • mL^{-1}		
Dose 1	2061 (18.5)	1883 (53.2)
Dose 2	2551 (20.1)	2769 (26.8)
Dose 3	2588 (30.1)	2800 (26.8)
Dose 4	3089 (16.9)	3626 (20.2)
AUC_{0-12h}, ng • h • mL^{-1}	10445 (19.3)	11390 (18.9)
AUC_{0-6h}, ng • h • mL^{-1}	4677 (18.2)	4809 (26.5)
AUC_{6-12h}, ng • h • mL^{-1}	5756 (21.5)	6484 (21.6)
C_{max}, ng/mL		
Dose 1	1502 (12.6)	1816 (24.8)
Dose 2	1668 (21.0)	1871 (30.8)
Dose 3	1326 (41.3)	1543 (34.4)
Dose 4	1711 (17.7)	2239 (26.5)
$C_{max0-12h}$, ng/mL	1883 (11.8)	2543 (9.73)
t_{max}, h		
Dose 1	0.333 (0.300-1.00)	0.650 (0.333-2.97)
Dose 2	0.500 (0-1.50)	0.500 (0-1.50)
Dose 3	1.00 (0.500-2.00)	1.32 (0.667-2.33)
Dose 4	0.500 (0.450-0.500)	0.983 (0.483-2.00)

Geometric mean (percentage coefficient of variation [CV%]) values shown for AUC and C_{max}.; median (range) values shown for t_{max}.
AUC = area under the curve; C_{max} = maximum observed plasma concentration; t_{max} = time to C_{max}.
